# Whole-Genome Shotgun Sequence of *Arthrospira platensis* Strain Paraca, a Cultivated and Edible Cyanobacterium

**DOI:** 10.1128/genomeA.00751-14

**Published:** 2014-08-07

**Authors:** Francois Lefort, Gautier Calmin, Julien Crovadore, Jacques Falquet, Jean-Pierre Hurni, Magne Osteras, Francois Haldemann, Laurent Farinelli

**Affiliations:** a Plants and Pathogens Group, Research Institute, Earth Nature and Environment, Hepia, University of Applied Sciences Western Switzerland, Jussy, Switzerland; b Faculty of Engineering and Architecture, University of Applied Sciences of Western Switzerland, Delémont, Switzerland; c JFS, Meyrin, Switzerland; d School of Geosciences and the Environment, University of Lausanne, Lausanne, Switzerland; e Fasteris SA, Plan-les-Ouates, Switzerland; f Biorigin SA, Meyrin, Switzerland

## Abstract

Here we report the whole-genome shotgun sequence of a Peruvian strain of *Arthrospira platensis* (Paraca), a cultivated and edible haloalkaliphilic cyanobacterium of great scientific, technical, and economic potential.

## GENOME ANNOUNCEMENT

*Arthrospira platensis*, a filamentous cyanobacterium, forms massive populations in tropical water bodies characterized by high levels of carbonate and bicarbonate and pH up to 11 (1). It is an edible microorganism whose consumption remains traditional in Chad (2). Well-documented nutritional and therapeutic properties of *A. platensis*, mainly as a tool against malnutrition (3), have elicited a growing market of several thousand tons yearly. As an extremophile photosynthetic microorganism, *A. platensis* is suitable for mass-production (4), including for the mass-production of highly valued biopolymers (5). The high pH (up to 11.5) of its fully mineral culture medium provides an efficient barrier against other bacterial contamination (6). Moreover, it is a strong candidate for life-sustaining systems in future spatial or submarine missions (7), and is also considered to be, along with other photosynthetic aquatic microorganisms, a potential biofuel source (8).

*Arthrospira platensis* strain Paraca was provided by Biorigin SA (Switzerland). We realized axenic cultures cultivated in a modified Zarrouk medium at 25°C (6). Cultures were checked for bacterial contaminations prior to DNA extraction, performed on a 100 mg (FW) filtered sampled according to a modified DNA extraction micro-method (9). Whole-genome shotgun sequencing of the Paraca strain was then carried out in an IIlumina HiSeq 2000, producing 22,924,753 paired-end reads 100 bp long. Assembling conducted with SPAdes v3.0 (10) led to 268 contigs for a genome length of 6,501,886 bp (contig *N*_50_ of 72,660 bp) with an average G+C content of 44.31%. Annotation through the Prokaryotic Genomes Automatic Annotation Pipeline Group (PGAAPG) predicted 5,824 genes including 5,439 protein coding sequences (CDs). It also identified 6 clustered regularly interspaced short palindromic repeat (CRISPR) arrays, 388 pseudogenes, and 47 RNA genes while RAST analysis (11) identified 7,605 CDs and 46 RNA genes (40 tRNA and 6 rRNA genes). A comparison of this sequence to previously registered sequences of *Arthrospira* genomes has been published (12). As observed in *Arthrospira* sp. PCC 8005 (13), numerous gene members of the same metabolic pathways are dispersed over the genome. Data analysis identified genes coding for hydrogenases involved in H_2_ production such as Hox and Hyp loci and 1 complete and 2 partial nitrogenase sequences. Regarding the nitrogen metabolism, this strain is equipped for nitrate and nitrite uptaking, ammonium synthesis, ammonium assimilation, and for nitrogen assimilation through a typical Mo-dependent nitrogenase.

Numerous genes involved in carotene, thiamine, tocopherol, biotin, cobalamin, and quinones metabolisms are present. The strain also possesses genes of resistance to semi-metals and metals such as arsenic, copper, cobalt, zinc, and cadmium as well as a mercuric ion reductase. No plasmid sequence or complete phage sequences were found, whereas a few interspersed prophage protein or plasmid stabilization protein sequences were present. Furthermore, no genes coding for known cyanobacterial toxins biosynthesis pathways were detected. An in-depth study of the genome of *A. platensis* should elucidate the mechanisms involved in this bacterium’s survival in hyperalkaline environments, and suggest possible industrial usages.

### Nucleotide sequence accession numbers.

This whole-genome shotgun project has been deposited at DDBJ/EMBL/GenBank under the accession no. ACSK00000000. The version described in this paper is version ACSK03000000 (GenBank Assembly ID GCA_000175415.3).
